# Burden of diseases and disease trends in the rehabilitation public health facilities in Gauteng Province, South Africa

**DOI:** 10.4102/hsag.v31i0.3511

**Published:** 2026-10-30

**Authors:** Sonti Pilusa, Khetsiwe Masuku, Simon Rabothata, Tshifhiwa Mukheli, Vaneshveri Naidoo

**Affiliations:** 1 Department of Physiotherapy, Faculty of Health Sciences, University of the Witwatersrand, Parktown, South Africa; 2 Department of Speech Therapy, Faculty of Humanities, University of the Witwatersrand, Braamfontein, South Africa; 3 Department of Health, Johannesburg, South Africa

**Keywords:** curriculum, rehabilitation, health information, South Africa, disability

## Abstract

**Background:**

Rehabilitation remains an unmet need and a neglected health service in South Africa. To address the country’s complex burden of disease, a skilled rehabilitation workforce is needed.

**Aim:**

This study aimed to identify prevalent medical conditions and disease trends among patients treated by rehabilitation therapists over a six-year period. It further examined whether these prevalent health conditions are represented in the rehabilitation health education curricula.

**Setting:**

The study was undertaken in public healthcare facilities within Gauteng province, South Africa.

**Methods:**

Secondary data on provincial rehabilitation data from April 2019 to March 2024 were analysed and categorised into non-communicable diseases (NCDs), children-specific conditions, injuries, and communicable diseases. Data were analysed descriptively. Lastly, the undergraduate rehabilitation health training curriculum (Occupational therapy, Physiotherapy, Speech therapy and Audiology) was reviewed to assess whether it covers the medical conditions treated by rehabilitation therapists.

**Results:**

The most treated conditions were NCDs (44.1%), injuries (36.4%) and children-specific conditions (15.6%). All health conditions decreased during the coronavirus disease 2019 pandemic, followed by an increase in NCDs and injuries between 2022 and 2024. The undergraduate rehabilitation health training curriculum covers the most prevalent conditions treated by public sector rehabilitation therapists.

**Conclusion:**

The burden of diseases treated in public healthcare rehabilitation departments aligns with the national burden of diseases. The impact of the COVID-19 pandemic on rehabilitation services is evident in the trend analysis over the years. Continual review of the curriculum is needed to ensure local health responsiveness.

**Contribution:**

This study highlights the burden of disease treated by the public health sector rehabilitation therapists, the trends over time and the importance of ensuring that the curriculum is locally responsive.

## Introduction

### Background

For years, the quadruple burden of disease, made up of HIV, non-communicable diseases (NCDs), injuries and maternal and child health, has been a challenge for the South African health system (Mayosi et al. 2012). This challenge is worsened by the rising burden of multimorbidity in a health system that is already overburdened and struggling to ensure adequate health care for all (Maphumulo & Bhengu 2019; Roomaney et al. 2021). Another concern in the South African health system is the neglect of the burden of disability because of chronic conditions (Carpenter, Nyirenda & Hanass-Hancock 2022; Charumbira, Berner & Louw 2022; Myezwa et al. 2018). The total Years Lived with Disability (YLDs) was estimated at 6.50 million in 2019; the estimate doubled from 1990 because of the ageing population and a high burden of chronic diseases such as HIV, NCDs and musculoskeletal conditions (Achoki et al. 2022).

The rise in chronic conditions increases the burden of disability, necessitating rehabilitation care (Charumbira et al. 2022; Myezwa et al. 2018). A scoping review by Charumbira et al. (2022) reported on the functional problems associated with the top 10 health conditions contributing to years of disability in South Africa. The health conditions were HIV, tuberculosis (TB), chronic lung diseases, type 2 diabetes, hearing loss, musculoskeletal problems, neonatal-related health conditions, fractures, cardiovascular diseases, strokes, and burns (Charumbira et al. 2022). The most reported functional problems associated with the top 10 health conditions were mobility problems, pain, and mental health problems (Charumbira et al. 2022). Louw et al. (2021) reported on the common rehabilitation-amenable conditions based on the Global Burden of Disease in 2017 and projections for 2022. The conditions that could be addressed by rehabilitation included HIV, non-communicable diseases (cardiovascular disease & heart failure, cardiovascular accidents and diabetes), injuries (burns and Fractures and dislocations); children-related disorders (neonatal disorders and congenital birth defects); hearing loss; musculoskeletal disorders; and chronic respiratory diseases. The conditions that had a high projected percentage increase from 2017 to 2022 were cardiovascular (5.3%), diabetes (1.3%), burns (0.8%), hearing loss (4.1%), neonatal disorders (15.9%) and musculoskeletal problems (3.9%) (Louw et al. 2021). These predictions can guide rehabilitation service planning and rehabilitation higher education training (Louw et al. 2021).

Rehabilitation plays a vital role in disability management. Rehabilitation entails interventions that focus on enhancing functional ability and quality of life in people with chronic conditions and those with disabilities (World Health Organization [WHO] 2017b). The WHO has highlighted the need to strengthen rehabilitation care by 2030 and pointed out how rehabilitation is needed to promote healthy lives and well-being for all at all ages, as articulated by Sustainable Development Goal (SDG) number three (Cieza 2019; WHO 2017b). Despite these global initiatives, there are multiple challenges in providing sufficient rehabilitation treatments in South Africa. For example, the healthcare system tends to be more curative and reactive, neglecting services that can improve functional outcomes (Pilusa, Myezwa & Potterton 2021). There is poor access to rehabilitation services, assistive device procurement difficulties, shortage of the rehabilitation workforce and inefficient rehabilitation health information system (Louw et al. 2023; Morris et al. 2019). These challenges highlight the urgency to strengthen rehabilitation care to support the ‘Rehabilitation 2030’ global call (WHO 2017a).

Rehabilitation health information is one of the building blocks of a health system that needs attention in South Africa (Morris et al. 2019). The South African national health indicators that are routinely collected do not include long-term care needs for people with chronic conditions and disability-related data (Louw et al. 2023; Ntsiea et al. 2017). Data on population functioning, sequelae of chronic conditions, and rehabilitation needs are scarce in the national health indicators, even though the health system is overburdened with the quadruple burden of diseases that contribute to disability (Louw et al. 2021). The only national indicator that speaks to rehabilitation is assistive device provision (Louw et al. 2023). Poor rehabilitation health information data management will continue to undermine the long-term needs of people with chronic conditions and people already living with disabilities. In addition, without adequate health information, planning for rehabilitation service delivery and the needed resources will be undermined.

To achieve the global and local rehabilitation goals, rehabilitation workforce shortages and training must be addressed. There is a shortage of the rehabilitation workforce in South Africa (Louw et al. 2023; Morris et al. 2019; Tiwari, Ned & Chikte 2020). In 2019, there were 3519 rehabilitation therapists in the public sector (1504 physiotherapists, 1279 occupational therapists and 730 speech therapists and audiologists). To meet international rehabilitation workforce standards, 42 254 rehabilitation therapists are needed in the public health sector (Tiwari et al. 2020). Thus, there is a need to graduate more skilled rehabilitation professionals who can meet the local health needs.

Rehabilitation Initiative Priority area 6 proposes ‘Developing a strong multidisciplinary rehabilitation workforce that is suitable for the country context’ (WHO 2017a). To ensure that rehabilitation needs are not overlooked and to foreground priority area 6, higher education rehabilitation curricula require interrogation and comparison of content to the most prevalent medical conditions treated by rehabilitation therapists. One element of workforce development to meet these goals is education through curricula in higher education institutions. This study expands on prior work to quantify the frequency of conditions treated by physiotherapists in state health facilities and alignment with the curriculum (Ntsiea et al. 2017). Ntsiea et al. (2017) found that the most prevalent conditions treated by physiotherapists were injuries and strokes, and there was alignment between what was treated in the public health facilities and what was covered in the curriculum. Therefore, this study aimed to identify the most prevalent medical conditions and the disease trends over 6 years treated by public health sector rehabilitation therapists. Secondly, to establish whether the most prevalent conditions are covered in the rehabilitation health education curriculum.

## Research methods and design

### Study design

The authors used a mixed study approach incorporating a retrospective design and a document review. A retrospective design was used to analyse the health conditions treated in public healthcare rehabilitation departments in Gauteng province, as well as disease trends over 6 years. A document review was conducted on the curriculum documents.

### Study setting

The study was conducted in Gauteng province, the smallest and most populated province, because it is the economic hub of the country (Statistics South Africa 2024). Gauteng province comprises three metropolitan municipalities: the City of Ekurhuleni, the City of Johannesburg, and the City of Tshwane, and two district municipalities.

The Gauteng Department of Health (GDoH) rehabilitation services are integrated within 43% (169/372) of primary health facilities, 12 district hospitals, three tertiary hospitals, nine regional hospitals, four central hospitals and five specialised hospitals, namely the Psychiatric Hospital, TB Hospital, Rehabilitation Hospital and mental healthcare rehab hospital.

Rehabilitation services rendered in the province include Speech Therapy, Audiology, Occupational Therapy and Physiotherapy. There are additional services such as podiatry, orthotics and prosthetics under a separate sub-directorate.

Our second study site was at a higher education institution in Gauteng province. This higher education institution offers Physiotherapy, Occupational Therapy, Speech Therapy and Audiology programmes (undergraduate degree training).

### Data source

Data for the record review were sourced from the GDoH rehabilitation services. Gauteng province health information data on the rehabilitation database was established in 2019 to improve monitoring and evaluation of rehabilitation services’. Public health rehabilitation service managers within the GDoH facilities at all levels of care (primary health, district health and tertiary health levels) in the province submit monthly data on services rendered on services rendered, health conditions/diseases treated, duration of the treatment and number of treatment sessions. The period of analysis was from 2019–2024 to show the rehabilitation demand before and after the coronavirus disease 2019 (COVID-19) pandemic.

The monthly data on rehabilitation in the province were captured and submitted on a Microsoft Excel spreadsheet. Data were cleaned and repeated health conditions were removed. Categories labelled ‘other’ in the data set were removed. Data were categorised into broad disease headings, namely non-communicable diseases, injuries, communicable diseases, and children-related conditions. Categorisation of the diseases was conducted by SP, KM and VN. SR and TM confirmed the data and some of the abbreviated health conditions.

The second data source was the undergraduate curriculum documents (discipline-specific curriculum documents and the medicine and surgery curriculum), which outline health conditions and diseases taught in the undergraduate teaching programmes in Physiotherapy, Occupational Therapy, Speech-Language Pathology and Audiology departments at the university where the research was conducted. The curricula data were captured on an Excel spreadsheet. The information was configured into the quadruple burden of disease headings: HIV and AIDS and TB; non-communicable diseases; injury; maternal and child health, as well as mental health and a category for other (Online Appendix 1: Addendum 5 Table 1-A5). The curricula were independently reviewed by two researchers (SP, VN), and the data captured by the two researchers were compared for reliability. The curriculum was only reviewed for content and not how it is taught or assessed.

### Data analysis

Descriptive statistics, frequencies and percentages were used to analyse the health conditions treated by the rehabilitation therapists. All the health conditions that were < 1% were excluded (Online Appendix 1: Addendum 1, Addendum 2, Addendum 3 and Addendum 4). A comparison was made between what is offered in the curriculum and the data gathered from the GDoH rehabilitation departments.

### Ethical considerations

Ethical clearance was granted by the University of the Witwatersrand, with ethical clearance no. M230315 M230913-A-0001. The provincial health department granted permission to access the rehabilitation data. Permission to review the curricula was granted by the respective heads of departments.

## Results

The results demonstrate the number of common health conditions (Figure 1) treated by rehabilitation therapists over 4 years (2019–2024). Of the total health conditions, the most common conditions treated were non-communicable diseases (43.9%), followed by injuries (36.4%), and children’s specific conditions (16.5%). Figure 1 illustrates the burden of disease treated by the rehabilitation therapists.

**FIGURE 1:**
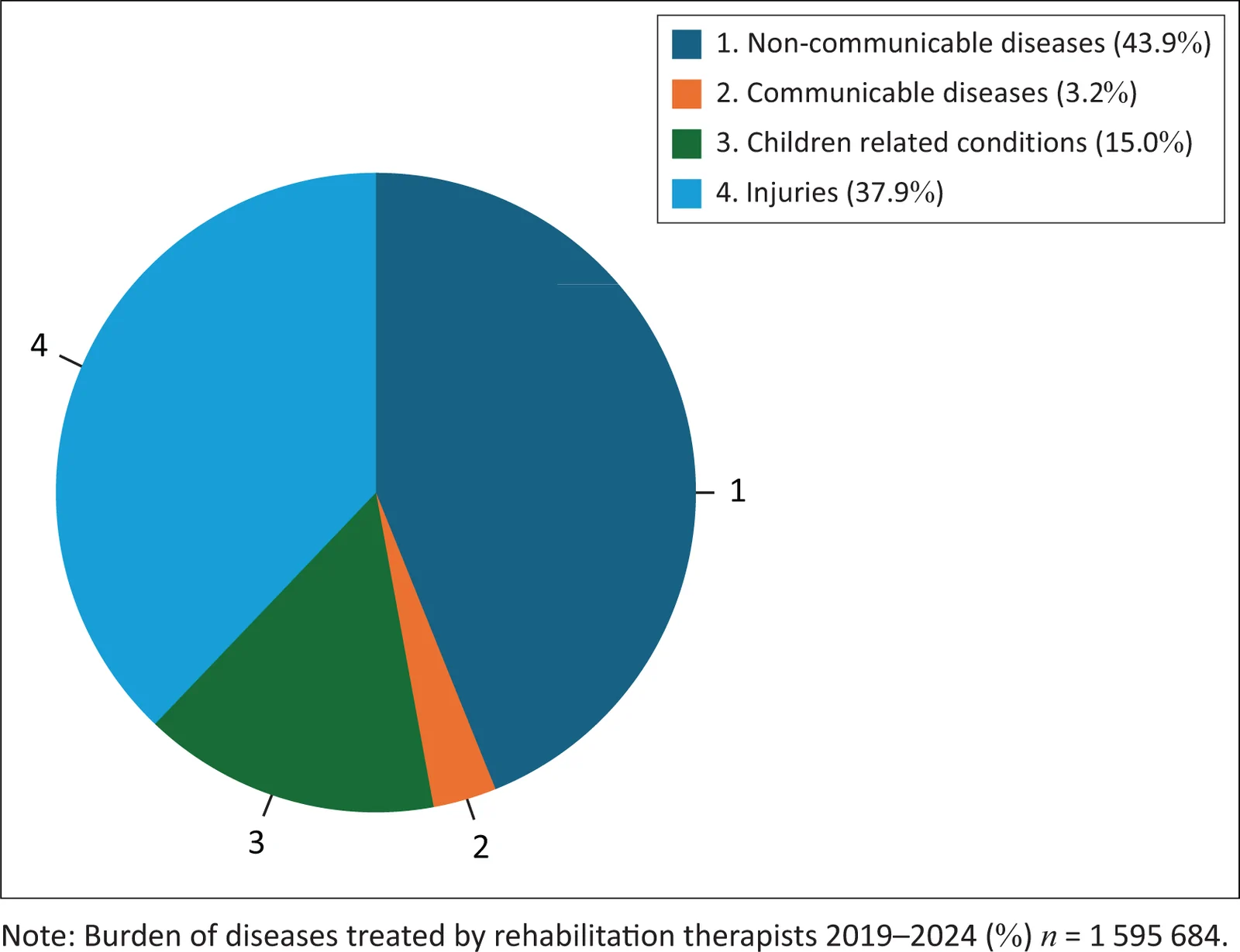
Burden of disease treated by rehabilitation professionals.

Figure 2 displays the disease trend between 2019 and 2024. Looking at the trends over the years, data from rehabilitation healthcare professionals on non-communicable diseases revealed a steady decline from the reporting period, 2019–2020 (45.3%) to the reporting period, 2021–2022 (41.8%). There was a gradual increase to 47.1% in the 2023–2024 reporting period.

**FIGURE 2:**
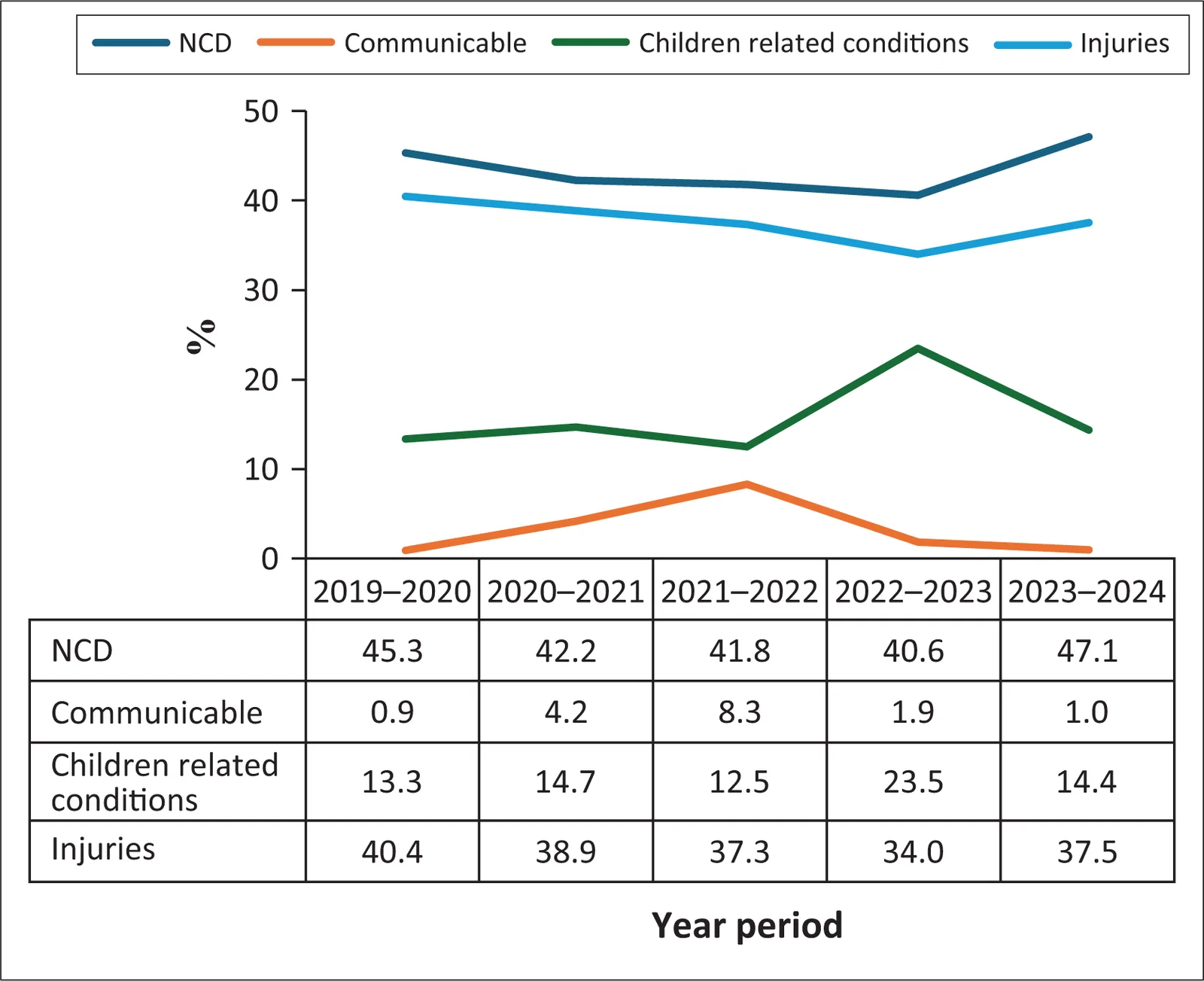
Disease trend over 4 years (2019–2024).

Injuries were 40.4% in the reporting period 2019–2020. A steady decline was observed during reporting periods 2021–2022 and 2022–2023.

Data on children-specific conditions were steady between 2019 and 2022, with a huge increase between 2022 and 2023 from 12.5% to 23.5%.

Communicable diseases have been significantly lower than other diseases in the rehabilitation healthcare data in public hospitals in Gauteng province. However, in the reporting periods 2020–2021 and 2021–2022, there was a significant increase, peaking at 8.3% in the reporting period 2021–2022.

### Non-communicable diseases

The top six prevalent NCDs were cardiovascular accident (26.2%), respiratory problems (21.9%), arthritis (11.9%), mental health problems (11.5%), back pain (9.1%) and cancer (6.6%). Table 1 outlines the types of NCDs.

**TABLE 1:** Non-communicable diseases (*N* = 626 093).

Health conditions	*n*	%
Cardiovascular accidents	164 209	26.2
Respiratory difficulties	136 994	21.9
Arthritis	91 369	14.6
Mental health problems	71 877	11.5
Back pain	56 701	9.1
Cancer	41 427	6.6
Drug overdose	24 579	3.9
Amputation (vascular conditions)	22 937	3.7
Hypertension	9095	1.5
Epilepsy	6905	1.1

**Total**	**626 093**	**100.0**

### Injuries

Soft tissue injuries (70.2%) were the most prevalent injuries, followed by burns (13.4%), as illustrated in Table 2.

**TABLE 2:** Types of injuries (*N* = 66 3019).

Health conditions	*n*	%
Soft tissue, fractures, nerve injuries (upper and lower limb)	465 544	70.2
Burns	88 593	13.4
Traumatic brain injury	47 216	7.1
Spinal cord injuries	44 647	6.7
Amputations (upper and lower limb)	17 019	2.6

**Total**	**663 019**	**100.0**

### Children-related conditions

Developmental delay (32.2%) and cerebral palsy (25%) were the most prevalent conditions among children, as presented in Table 3.

**TABLE 3:** Children-related conditions (*N* = 214 617).

Health conditions	*n*	%
Developmental delay	69 036	32.2
Cerebral palsy	53 687	25.0
Neonatal complications	16 234	7.6
Prematurity	13 616	6.3
Hypoxic-ischemic encephalopathy	10 427	4.8
Hydrocephalus	7599	3.5
Developmental drug overdose with onset in childhood or adolescence	6909	3.2
Other neonatal high-risk	6200	2.9
At risk (NOT neonatal risk)	5867	2.7
Mental retardation	4991	2.3
Poor school performance	4643	2.2
Genetic disorder	3649	1.7
Down syndrome	3680	1.7
Severe acute malnutrition (SAM)	3018	1.4
Behaviour and emotional drug overdose onset of child/adolescent – conduct, tic, attention, and activity	2528	1.2
Club feet	2533	1.2

**Total**	**214 617**	**100.0**

## Communicable diseases

Coronavirus disease 2019 (70%) was the most prevalent communicable disease treated between 2019 and 2024 as presented in Table 4.

**TABLE 4:** Types of communicable diseases (*N* = 50 972).

Health conditions	*n*	%
COVID-19	35 742	70.1
TB	5457	10.7
HIV and AIDS	4474	8.8
Meningitis	3969	7.8
Encephalitis	757	1.5
Congenital infection	573	1.1

**Total**	**50 972**	**100.0**

COVID-19, coronavirus disease 2019; TB, tuberculosis.

### Undergraduate rehabilitation curriculum

The undergraduate-level curriculum content (topics covered) for Physiotherapy, Occupational Therapy, Speech-language Pathology and Audiology at a higher education institution (HEI) in SA are outlined in Online Addendum 5 Table 1-A5. The authors found that there is no standard rehabilitation curriculum and each rehabilitation discipline emphasises different health conditions. The most common conditions seen in the public healthcare facilities are taught in the curricula for Physiotherapy, Occupational Therapy, Speech-Language Pathology and Audiology. The review did not investigate how the topics are covered and assessed. All the outlined topics cover the students learning the theory of pathology in medicine and surgery and in their discipline-specific roles, linking the topics to disability management. The students are examined on these topics in their theory and in clinical practice. Health promotion is taught in the four disciplines, covering disease prevention and public health principles.

## Discussion

To the authors’ knowledge, this is the first study to identify the most prevalent medical conditions and disease trends over 6 years, treated by public health sector rehabilitation therapists, and establishing whether the prevalent conditions treated are covered in the rehabilitation health education curriculum. The authors found the conditions treated by the rehabilitation therapists were NCDs, injuries (soft tissue injuries, burns and fractures), communicable diseases (HIV, TB and COVID-19) and children-related disorders. This finding aligns with the reported health conditions that are amenable to rehabilitation care and that contribute to the burden of disability in South Africa (Louw et al. 2021). The multiple impairments and functional problems that are associated with living with an injury or chronic condition underscore the need to incorporate rehabilitation services in chronic care management (Charumbira et al. 2022; Louw et al. 2021).

It was not surprising that NCDs (cardiovascular diseases, respiratory diseases, cancer, and mental health problems) were in the top five prevalent conditions treated in the rehabilitation departments. The prevalence of NCDs is high in low- and middle-income countries because of rapid unplanned urbanisation, globalisation of unhealthy lifestyles, population ageing and poverty. NCDs are reported to have overtaken HIV and TB in sub-Saharan Africa (Gouda et al. 2019). Disability-adjusted life-year (DALY) linked to NCDs was 151.3 million in 2017 compared to 90.6 million in 1990, with cardiovascular diseases, cancer and mental health heavily contributing to the NCD burden in sub-Saharan Africa (Gouda et al. 2019). Non-communicable diseases are the leading cause of death and disability in South Africa, with the top five contributing conditions being cardiovascular diseases (CVDs), cancer, type 2 diabetes mellitus, respiratory illnesses, and mental health disorders (Nolijana et al. 2016). Even with the disease trends over the 6 years, NCDs were on the increase. The increase in NCDs such as cardiovascular problems and hearing loss was also predicted by Louw et al (2021). As such, prevention strategies for NCDs, health promotion campaigns, encouraging physical activity, and smoking cessation must be part of rehabilitation clinical practice and curriculum.

Communicable diseases were the least treated conditions in the rehabilitation units. This is surprising given the impact of HIV on functioning and the increase in multimorbidity patterns of hypertension and HIV (Myezwa et al. 2018; Roomaney et al. 2021). This could mean that when capturing patients’ information in the rehabilitation health information system, only the primary health condition was captured, and co-morbidities were excluded (Roomaney et al. 2021). The implications of capturing the primary health conditions only will limit data on multimorbidity and the required service delivery planning and resources (Roomaney, van Wyk & Pillay-van Wyk 2023). Not surprisingly, COVID-19 was the highest treated communicable disease given the outbreak. The impact of COVID-19 on health services must not be underplayed. From 2020–2021, the reported health conditions were low, implying reduced utilisation of healthcare during the lockdown period because of fear around COVID-19. Disability related to COVID-19, termed long COVID, was not captured (Brown et al. 2020). Long COVID is an evolving health challenge that needs attention in terms of long-term monitoring of patients and research (Mendelson et al. 2021).

For any health system to function effectively, relevant health information is needed. Currently, the national and provincial health monitoring indicators do not include rehabilitation data, except for assistive device information (Louw et al. 2023). Rehabilitation information indicators are needed to estimate the need for rehabilitation services and to plan needed resources. Also, the rehabilitation health information data management system (data capture, analysis and usage) needs to be reviewed. Currently, it focuses on single diseases, missing multimorbidity data and disability related data. Such information is important given the rise in multimorbidity and disability from NCDs and communicable diseases in South Africa (Roomaney et al. 2021). Efforts to strengthen the rehabilitation health information system can contribute to promoting wellness beyond diagnosis and achieving global health initiatives such as SDG 3 and WHO Rehabilitation 2030.

The rehabilitation therapy curriculum covers topics on NCDs, injuries, communicable diseases and children-related conditions. The rehabilitation curriculum covers the conditions reported in the Global Burden of Disease that are amenable to rehabilitation (Cieza et al. 2020; Louw et al. 2021). The topics in the curriculum align with Sustainable Development Goal 3 ‘to ensure healthy lives and promote well-being for all at all ages’, targeting maternal health, NCDs, children’s health, injuries and communicable diseases. (WHO 2017b, 2017c). Through teaching specific health conditions in combination with population health, health promotion, disease prevention and holistic patient approaches, the curriculum is developing a rehabilitation cadre that contributes to achieving SDG 3 and the Rehabilitation 2030 (Pilusa et al. 2024).

The authors found that most of the conditions seen in the rehabilitation units or departments are covered in the undergraduate rehabilitation curricula. However, the content, format of teaching and assessments were not reviewed in this study. Future research can explore the content, format of teaching and assessment to ensure UG rehabilitation therapy graduates are skilled to manage local health needs. Tiwari et al. (2020) underscored the importance of reviewing the rehabilitation curriculum and adopting an interdisciplinary rehabilitation curriculum that is locally responsive to local health needs. The curriculum was silent on COVID-19. The curriculum needs to ensure that the long-term effects of COVID-19, ‘long COVID’, such as fatigue, respiratory problems, pain, muscle weakness and mental health problems, are included as more evidence on long COVID and disability emerges.

Higher education institutions’ curricula must be responsive to local health needs, considering the local health system constraints. Although rehabilitation graduates are skilled to manage many health diseases, limitations in the health system can hamper rehabilitation productivity. Challenges such as shortages of healthcare professionals, poor access to long-term rehabilitation in the community setting, limited integration of rehabilitation care at the primary level, and lack of adequate rehabilitation infrastructure can influence service delivery (Louw et al. 2023; Morris et al. 2019). The availability and distribution of rehabilitation therapists between the professions in the public health facilities should be considered as well (Tiwari et al. 2020). The availability and distribution of healthcare workers influence the availability of services that can be rendered and the underutilisation of skilled human resources. Hence, continual engagement between the rehabilitation training department and the public health systems is needed to address these challenges.

### Limitations

Firstly, the rehabilitation data-capturing process for the four disciplines is not standardised; thus, each discipline captured health data differently. Secondly, only one discipline adopted the quadruple burden of disease categories in the way they captured the health data. Although this approach is good, it limits data capture to single diseases and multimorbidity. Some disciplines used abbreviations instead of the correct medical terms, requiring the researchers to find out from the unit managers what the abbreviations stand for. Lastly, essential information such as demographic data (gender and age), risk factors and the presence of comorbidities was missing. The authors underscore the importance of the rehabilitation health information system in capturing, analysis and its usage.

The rehabilitation information system needs to be standardised, and the information needs to be regularly checked to ensure usability in planning for service delivery.

Related to the curriculum review, we acknowledge methodological limitations. The authors did not outline the content of the topics, how they are taught, the extent of clinical exposure of students to the different conditions, and assessment alignment.

### Recommendations

The rehabilitation data-capturing process for the four disciplines is not standardised. Therefore, a standardised electronic data-capturing system for rehabilitation data is recommended. A live electronic data system will ensure data are captured correctly and can be used to inform planning for the rehabilitation services in Gauteng province.

The authors did not outline the content of the topics, how they are taught, the extent of clinical exposure of students to the different conditions, and assessment alignment. Thus, the authors recommend future research on the rehabilitation curriculum review to explore responsiveness to the burden of disease in terms of teaching content, clinical exposure and assessment alignment. A participatory action research study will allow for stakeholder engagement on the curriculum review process.

## Conclusion

This study has given insight into the burden of diseases treated by rehabilitation care departments in Gauteng province. The results are in line with global trends, in which NCDs top the burden of disease list. This means planning for health services and allocation of resources must be informed by data such as these for improved health outcomes. The data presented raise awareness of the role of rehabilitation care in chronic care management. The impact of the COVID-19 pandemic on rehabilitation services is evident in the trends analysis over the years. Rehabilitation curricula covered the diseases that were treated in the rehabilitation department. Continual review of the curriculum is needed to ensure local health responsiveness.
